# Non-Invasive Microbial Metabolic Activity Sensing at Single Cell Level by Perfusion of Calcein Acetoxymethyl Ester

**DOI:** 10.1371/journal.pone.0141768

**Published:** 2015-10-29

**Authors:** Christina E. M. Krämer, Abhijeet Singh, Stefan Helfrich, Alexander Grünberger, Wolfgang Wiechert, Katharina Nöh, Dietrich Kohlheyer

**Affiliations:** IBG-1: Biotechnology, Forschungszentrum Jülich GmbH, Jülich, Germany; University of Freiburg, GERMANY

## Abstract

Phase contrast microscopy cannot give sufficient information on bacterial metabolic activity, or if a cell is dead, it has the fate to die or it is in a viable but non-growing state. Thus, a reliable sensing of the metabolic activity helps to distinguish different categories of viability. We present a non-invasive instantaneous sensing method using a fluorogenic substrate for online monitoring of esterase activity and calcein efflux changes in growing wild type bacteria. The fluorescent conversion product of calcein acetoxymethyl ester (CAM) and its efflux indicates the metabolic activity of cells grown under different conditions at real-time. The dynamic conversion of CAM and the active efflux of fluorescent calcein were analyzed by combining microfluidic single cell cultivation technology and fluorescence time lapse microscopy. Thus, an instantaneous and non-invasive sensing method for apparent esterase activity was created without the requirement of genetic modification or harmful procedures. The metabolic activity sensing method consisting of esterase activity and calcein secretion was demonstrated in two applications. Firstly, growing colonies of our model organism *Corynebacterium glutamicum* were confronted with intermittent nutrient starvation by interrupting the supply of iron and carbon, respectively. Secondly, bacteria were exposed for one hour to fatal concentrations of antibiotics. Bacteria could be distinguished in growing and non-growing cells with metabolic activity as well as non-growing and non-fluorescent cells with no detectable esterase activity. Microfluidic single cell cultivation combined with high temporal resolution time-lapse microscopy facilitated monitoring metabolic activity of stressed cells and analyzing their descendants in the subsequent recovery phase. Results clearly show that the combination of CAM with a sampling free microfluidic approach is a powerful tool to gain insights in the metabolic activity of growing and non-growing bacteria.

## Introduction

Metabolic activity is a very important parameter when analyzing prokaryotes, under starvation stress, exposed to antimicrobials or fed with alternative carbon sources for fermentation processes [1–5]. Nutrient limitations or dynamically changing environments provoke evolutionary optimised adaption of the bacterial metabolism to ensure survival of the species. Therefore, bacteria are capable of rapidly sensing important intrinsic and extrinsic parameters affecting their survival, growth, and reproduction. However, the term “metabolic activity” is a resulting sum parameter of many enzymatic reactions. Generally, metabolic activity can be determined by measuring a specific substrate conversion, a detectable enzyme activity or a metabolite [6].

In contrast to invasive metabolic activity measurements using, *e*.*g*. quenching, cell lysis, or harmful environmental changes, common strategies for non-invasive measurement of metabolic activity use fluorescence to resolve intracellular, metabolic pathways. Since the metabolic pathways vary between organisms as well as individuals, metabolic activity measurements have to be tailored to metabolic specificities of microorganisms. The sensing-regulation function of cells can be exploited by using biosensor constructs for which the host has to be genetically modified beforehand. An overview of biosensor applications is given in [7]. However, this is not a suitable approach if genetic modification is problematic, *e*.*g*. for environmental studies or fermentative production validation.

The differentiation in co-factor dependent metabolic processes like pump activity and co-factor independent enzymatic conversion by hydrolases and dehydrogenases is stressed in [8]. Thus, fluorescence can also be introduced by fluorogenic substrates inside cells. An overview about those substrates used for bacterial differentiation is given in [9,10]. Enzymatic hydrolysis of fluorogenic esters has been reported already for five decades to study living cells [11]. The commercially available CAM green is conventionally used, besides other fluorochromes, to validate viability of mammalian cells [12–14]. Intracellular carboxylesterase hydrolyses the ester groups of CAM to the corresponding carboxyl groups and alcohol is cleaved off equimolar [14]. The fluorescent product calcein is retained inside the cell due to its ionized carboxyl groups [15]. A recent study on cancer cells reported that the fluorochrome is transported out in an energy-dependent mechanism in dependency of environmental conditions of the cell. In general, cancer cells use ATP-binding cassette (ABC) transporters, *e*.*g*. MRP-1, which is related to the potential of multidrug resistance, to shuttle calcein through the membrane. The hydrolysis of CAM and the efflux of calcein is already commercialized for mammalian viability testing or used for testing of multidrug resistance potential of tumor cell lines [12,13].

Nevertheless, in the past green fluorescent calcein has also been presented for microbial analysis with flow cytometry devices [16,17]. Hitherto, calcein fluorescence signals were not reported for all bacterial strains [16]. However, gram positive *Staphylococcus aureus* cells analyzed by FACS exposed to heating and antimicrobials after 1 h incubation at optimal growth temperature showed significant differences of fluorescence compared to control measurements [18]. FACS systems are widely established in all fields of microbiology for high-throughput single cell analysis [19]. However, cells have to be sampled from their environment of interest and can only be measured once, resulting in a snapshot view.

As a complementary technique, time-lapse microscopy offers a high temporal resolution long time analysis of single cells. Calcein green was used to demonstrate nutritional stress induced exchange of intracellular material between gram positive *C*. *acetobutylicum* and gram negative *D*. *vulgaris*. Nevertheless, controlled experimental conditions according to nutrient supply or stress conditions and analysis of statistically relevant cell numbers can be challenging during live cell microscopy [17,20]. Microfluidic devices developed in recent years offer well controlled environmental conditions that can be easily combined with fluorescence time lapse microscopy [21,22]. Thus, combination of microfluidic devices with automated time lapse imaging opens the gate for single cell observations with high temporal and spatial resolution of statistically significant cell numbers.

For sensing the dynamics of single cell esterase activity and fluorochrome pump activity of the gram positive representative microorganism *C*. *glutamicum*, a microfluidic method is presented. The intracellular hydrolysis of a CAM derivative and subsequent calcein efflux were unraveled in isogenic prokaryotes and their descendants of several generations. *C*. *glutamicum* is a relevant model organism to study dormancy as well as antibiotic tolerance or resistance, since it is related to pathogens like *Mycobacterium tuberculosis* or *Corynebacterium diphtheriae* [23,24]. Dormant cells are characterized by reduced metabolism, no cellular growth, and the absence of cell division. These bacteria have to be distinguished from cells with the fate to die by proving metabolic activity or resuscitation [25].

Continuously perfused CAM converted by *C*. *glutamicum* has been applied to distinguish non-viable cells from metabolically active but non-growing cells. For the first time to our knowledge, violet fluorescent calcein efflux by a prokaryote was determined. This was realized by using our microfluidic cultivation technology in which several hundred microbial microcolonies can be cultivated in cellular monolayers under constant environmental conditions [26,27]. This setup facilitates the analysis of dynamical intracellular heterogeneities of bacterial physiology in combination with high temporal resolution given by the combination of microfluidics and fluorescence time lapse microscopy [26].

## Material and Methods

### Microfluidic Device

**Fig 1:**
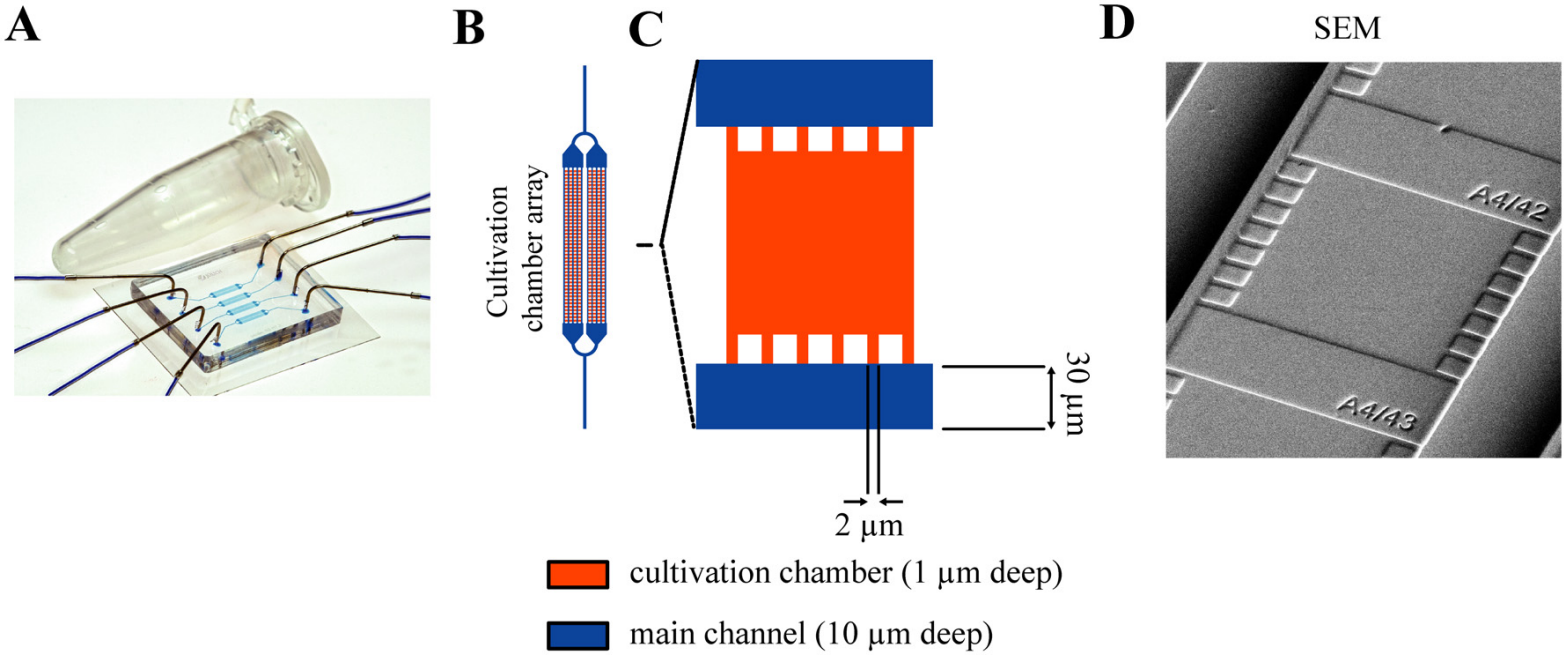
Microfluidic device and its submicrostructures. **(A)** Assembled microfluidic device with connected inlet and outlet tubings. **(B)** Parallel cultivation chamber arrays with branched main channels that subdivide in smaller channels for media perfusion. A a single device contains four cultivation chamber arrays with 352 chambers each. **(C)** Microcultivation chamber. **(D)** SEM micrograph of a cultivation chamber.

The microfluidic device as depicted in Fig 1A is based on common polydimethylsiloxane micro-molding as described in full detail by Grünberger et al. 2013 [28]. The picoliter sized micro-structured cultivation chambers have a height of 1 μm facilitating the growth of monolayered isogenic microcolonies. Several hundred cultivation chambers are arranged in parallel in four arrays for each of the four main channels (Fig 1B).

### Bacterial Cultivation

*Corynebacterium glutamicum* ATCC 13032 was pre-cultivated in a 20 mL shaking culture with brain heart infusion (BHI, BD, Heidelberg, Germany) at 30°C overnight. For microfluidic cultivation using complex medium BHI, another shaking flask culture with BHI was inoculated with the pre-culture. Exponentially growing cells of the shaking flask culture were transferred into the microfluidic device. Else two further pre-cultures were performed in minimal medium CGXII described by Keilhauer et al. 1993 containing 4% (w/v) glucose [29]. Media for microfluidic cultivations were modified as indicated by pH adjustment, omitted addition of glucose, protocatechuate, and iron, respectively, or addition of 10 μg/mL ampicillin or 10 μg/mL chloramphenicol, respectively. A constant cultivation temperature of 30°C was ensured for microfluidic cultures by an incubation chamber (PeCon GmbH, Erbach, Germany) mounted on the inverted epifluorescence microscope.

### Fluorescence Time Lapse Imaging

The microfluidic device was installed on a fully motorized time-lapse epifluorescence microscope (NIKON TI-Eclipse) with thermal drift compensation (Perfect Focus System) and a Plan Apo 100 Oil Ph3 DM objective. All microscope components were purchased from Nikon GmbH, Düsseldorf, Germany if not specifically stated. The setup was further equipped with a digital CMOS camera (Neo sCMOS, Andor Technology Plc., Belfast, United Kingdom), white LED illumination (pE-100, CoolLED, Andover, UK) for phase contrast imaging and a mercury light source (NIKON Intensilight) for epifluorescence illumination. CAM was analyzed with a filter set (EX 340–380 nm, DM 400 nm, BA 435–485 nm). Dihydroxycalcein acetoxymethyl ester (DHCAM) was excited and imaged every 6^th^ frame using a filter set (EX 465–495 nm, DM 505 nm, BA 515–555 nm). The transient state of initial CAM uptake and conversion of freshly seeded bacterial cells during the experimental set-up phase were imaged at high temporal resolution (frame interval 2.4 sec). Elsewise, an imaging frame interval of 8 min was kept.

### Metabolic activity sensing method and experimental validation

The perfusion media for microfluidic cultivation contained 46.3 μM calcein acetoxymethyl ester (Thermo Fischer Scientific, Darmstadt, Germany), if not indicated else, and were infused at a rate of 300 nL/min with a high-precision syringe pump (neMESYS, Cetoni GmbH, Korbussen, Germany) through the microfluidic device during time lapse imaging. The rate of photobleaching was determined with growth inhibited cells under glucose free conditions by constant illumination with the same light intensity chosen for all experiments. Cells were grown overnight and perfused with CGXII + 4% glucose and CAM. The medium perfusion was stopped for several hours until the calcein fluorescence was constant before photobleaching was measured on 85 individual single cells in a fluorescence range from 110 AU to 700 AU. The intensity loss by photobleaching during light exposure was determined as percentage from the initial single cell mean fluorescence per frame. The presence of phototoxicity was determined by detection of radical oxygen species (ROS) with dihydroxycalcein acetoxymethyl ester (DHCAM). The influence of co-metabolization of the ester groups of CAM was analyzed using the CAM surrogate substrate methyl methoxyacetate. Three mol methyl methoxyacetate corresponded to one mol CAM.

### Data Analysis

Time lapse image data was analyzed with a customized workflow implemented as an ImageJ/Fiji plugin [30]. Cell identification was performed using a segmentation procedure specialized to detect individual bacteria in crowded populations. Detected cells were subsequently tracked throughout image sequences using an adapted single particle tracking approach as implemented in TrackMate [31]. The image analysis was used for extraction of area and fluorescence of individual cells, as well as derived quantities (*i*.*e*., mean fluorescence of all cells of a colony). The analysis and visualization software *Vizardous* [32] assisted with analysis and interpretation tasks of single cell data in an interactive, configurable and visual way by augmenting lineage trees with time-resolved cellular characteristics.

### Calculations

The apparent growth rate μ_app_ was calculated by the increase in the sum of cell sizes of a colony during 10 frames (80 min) in minimal medium CGXII and 6 frames (48 min) in complex medium BHI. Single cell reaction rate constants of conversion or efflux were determined by the course of the mean fluorescence directly after shift of media condition to carbon free medium (CGXII—GLC—PCA) and after backshift to CGXII + 4% GLC, respectively.

## Results

### CAM uptake and calcein fluorescence formation

A dynamic, non-invasive metabolic activity sensing method was set up successfully using the biotechnologically relevant model prokaryote *C*. *glutamicum* ATCC 13032. The esterase substrate CAM is taken up by the bacterium and converted intracellularly to violet fluorescent calcein. The mean single cell fluorescence of bacterial cells, which is the averaged fluorescence signal of all pixels belonging to an imaged cell, described the metabolic activity considered of esterase activity and calcein efflux of bacteria on single cell level. The average single cell fluorescence of all cells of a microcolony was designated as mean fluorescence.

**Fig 2:**
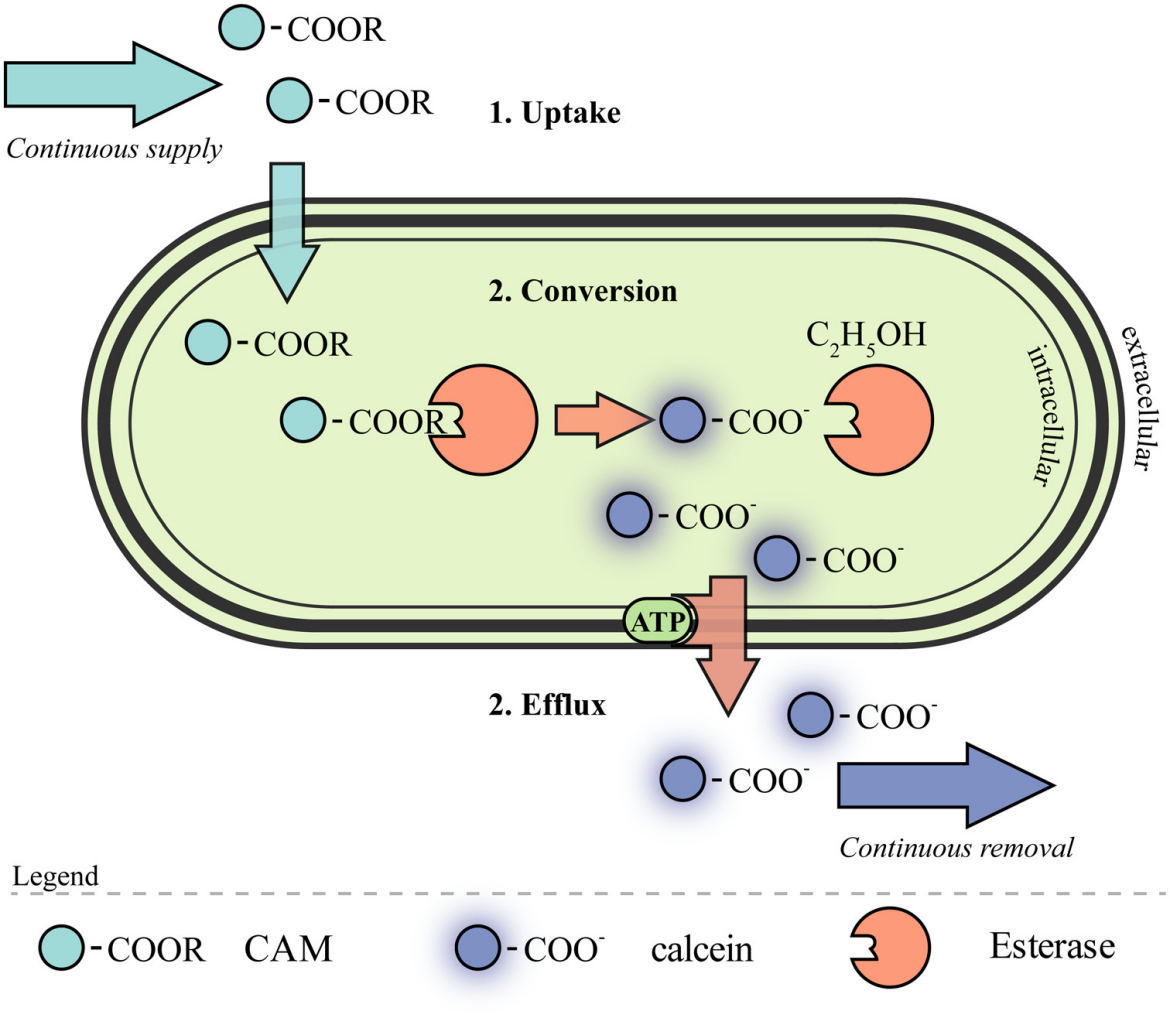
Cellular CAM metabolism model of a *C*. *glutamicum* cell. Gram positive *C*. *glutamicum* has a cell wall of different layers, which has to be passed by the fluorogenic substrate CAM. Once the cell wall is transversed by a presumably non-passive diffusive mechanism, CAM is converted to the fluorophore calcein and ethanol by intracellular carboxylesterases. The acidic calcein is assumed to be secreted by an energy dependent transport mechanism with a putative ATPase.

The mechanism how CAM passed the approximately 32 nm thick cell wall of four different layers and the cell membrane of the gram positive prokaryote is not unraveled yet [33]. However, the metabolic route of CAM and its hydrolysis product calcein is illustrated schematically in Fig 2. CAM is hydrolyzed intracellularly to its corresponding carboxylic acid and all ester groups are cleaved off and the fluorochrome accumulates in living cells. The acidic calcein is secreted partially according to the cells fitness and reaction to the environmental conditions.

CAM was fed in excess continuously together with complex medium (BHI) or minimal medium (CGXII) into a supply channel to feed single bacterial cells seeded into sequential cultivation chambers, followed by growth and division. Four cultivation conditions could be compared during every microfluidic cultivation in parallel.

S1 Video displays metabolic activity sensing of growing bacteria under reference conditions at pH 7. Generally, the mean single cell fluorescence of growing bacteria increased linearly over time until cell division. After cell division smaller daughter cells showed reduced mean single cell fluorescence compared to their mother cell.

**Fig 3:**
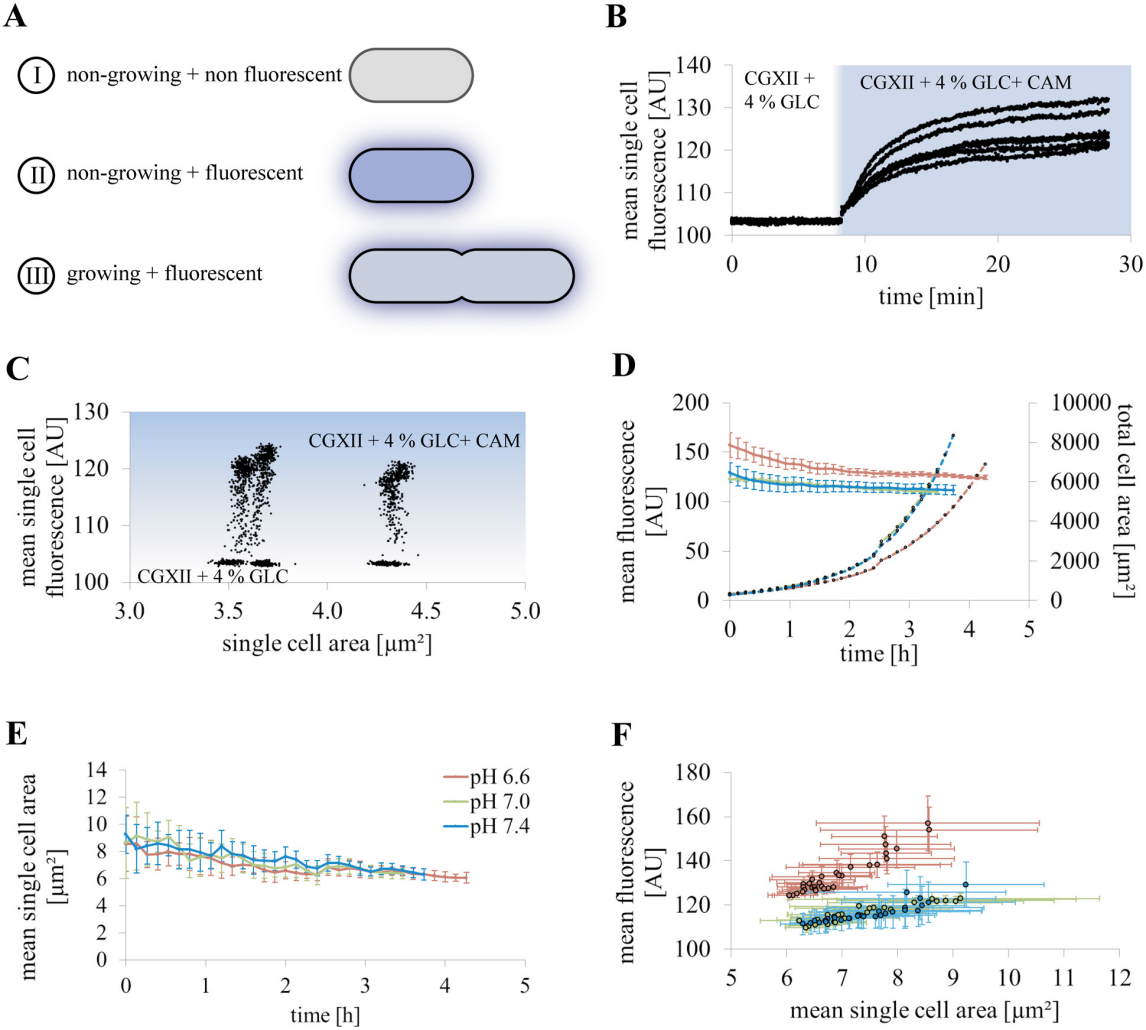
Metabolic activity sensing of growing and dividing cells. **(A)** Non-growing cells can be classified into non-viable non-fluorescent cells and non-growing but metabolically active bacteria, which showed highest mean single cell fluorescence in comparison. Growing bacteria showed moderate to medium fluorescence. **(B-C)** Mean single cell fluorescence of freshly seeded and heterogeneous sized bacteria is given after a change to minimal medium CGXII + 4% GLC containing CAM (n = 6 cells). **(B)** Increase in the mean single cell fluorescence of six individual cells of different cell size is shown over time after infusion of CGXII + 4% GLC + 46 μM CAM. **(C)** The mean single cell fluorescence of the individual cells presented in (B) is given according their size during the short term experiment. Increase of cell size due to growth could was neglectable during the experimental time of 30 min. Mean single cell fluorescence in dependency of cell size revealed marginal differences of the final equilibrium mean fluorescence of individuals. **(D-F)** Metabolic activity sensing of cells grown in complex medium BHI at pH 6.6 (red), pH 7.0 (green), and pH 7.4 (blue), respectively. **(D)** Average values of 10 cultivation chambers are presented. No significant difference in calcein fluorescence (solid lines) and growth represented by total cell area (dashed lines) could be observed for cultivation of *C*. *glutamicum* at pH 7.0 and pH 7.4. At pH 6.6, however, increased mean calcein fluorescence as well as a reduced total cell area was observed (n = 10 colonies analyzed at each pH). **(E)** The mean single cell area indicated a tendency of cell size reduction in average over time (n = 10 colonies analyzed at each pH). **(F)** Mean fluorescence correlates positively with the mean single cell area at all three pH values (n = 10 colonies analyzed at each pH).

Non-growing but viable cells exhibited higher mean single cell fluorescence than growing bacteria under comparable cultivation conditions. Cells considered as non-viable showed no significant mean single cell fluorescence. The schematic categories according to growth and metabolic activity are depicted in Fig 3A. The half time t_50_ of fluorescence signal formation in freshly seeded *C*. *glutamicum* cells following CAM uptake and subsequent enzymatic conversion was determined to be 10.6 ± 1.0 min in CGXII + 4% GLC. This was measured immediately after the addition of CAM to the medium (Fig 3B). The difference of endpoint mean single cell fluorescence of individual cells indicated that the CAM uptake was not strictly by passive diffusion (Fig 3B and 3C) as it is supposed for CAM green and mammalian cells [34].

Metabolic activity sensing was performed with complex medium BHI at media pH at 7.0 ± 0.4 to test the method under non-toxic cultivation conditions. *C*. *glutamicum* is reported to maintain its internal pH at 7.5 ± 0.5 in an external pH range of 6 to 9 [35]. Hence, the external pH was not supposed to alter the intracellular pH drastically circumventing detectable influence on calcein fluorescence. We determined that an external pH of 7.0 and higher had neither significant impact on the maximal growth rate (0.97 ± 0.03 h^-1^ at pH 7.0 and 1.02 ± 0.02 h^-1^ at pH 7.4, respectively) nor on the mean fluorescence over time. In contrast, at external pH 6.6 the mean fluorescence signal was clearly increased. Furthermore, a 20% decrease of maximal growth rate μ_max_ to 0.78 ± 0.02 h^-1^ due to a significant reduction of total cell area over time was observed (Fig 3D, S2 Video). The mean fluorescence of all three growth conditions showed an initial decline within the two hours (Fig 3D). This happened simultaneously with a decrease in the mean single cell area over time (Fig 3E). Due to the correlation of mean single cell area and mean fluorescence (Fig 3F), consequently the mean fluorescence decreased over time.

### CAM concentration optimization

**Fig 4:**
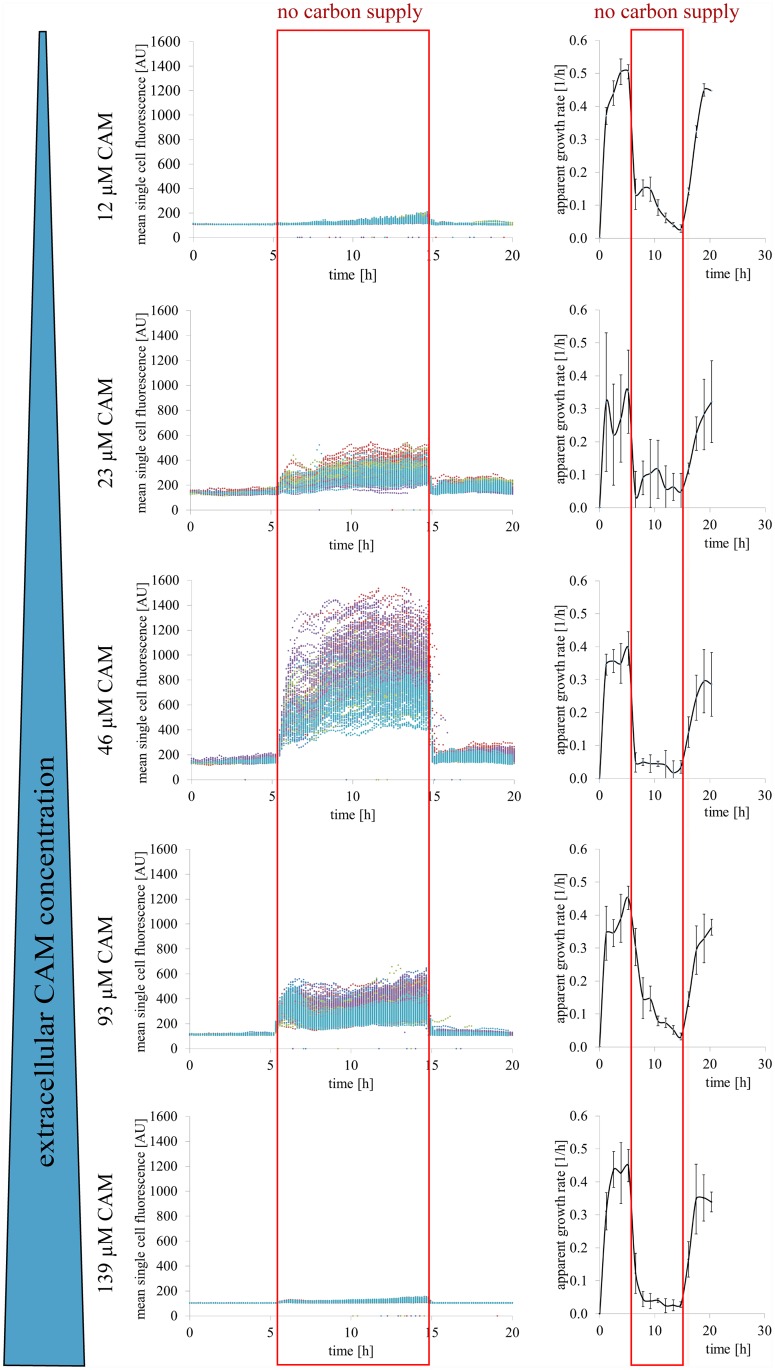
Comparison of mean single cell fluorescence and apparent growth rate at different extracellular CAM concentrations. Mean calcein fluorescence for five extracellular CAM concentrations are shown for five cultivation chambers each (every chamber is indicated with a separate colour). Perfusion medium was switched from CGXII + 4% GLC to carbon free CGXII medium (CGXII—PCA) supply for 10 h (indicated with red frame) to inhibit the energy-dependent calcein efflux. CAM conversion by intracellular esterase activity and subsequent calcein fluorescence showed a non-linear fluorescence increase except for concentrations higher than the optimal extracellular CAM concentration of 46 μM. The corresponding apparent growth rates changed according to the carbon supply and not because of an increase CAM concentration.

The CAM concentration in the perfusion medium was optimized to yield the highest fluorescence signal for online metabolic activity monitoring characterized by a hydrolysis reaction and a homeostasis like product efflux. Five different extracellular CAM concentrations (12 μM, 23 μM, 46 μM, 93 μM, or 139 μM) CAM in CGXII + 4% GLC (feast condition) and in despite of CAM carbon free CGXII—PCA (famine condition) were prepared, respectively (Fig 4). The perfusion of *C*. *glutamicum* cells with these CAM concentrations showed a significant increase in the mean single cell fluorescence during a famine phase of 10 h. For CAM concentrations higher than the optimal concentration at 46 μM no further increase in the mean single cell fluorescence could be observed. Instead, the mean single cell fluorescence decreased of CAM concentrations above 93 μM. The increase in the mean single cell fluorescence within the indicated starvation phase was followed by a sudden decrease after re-providing carbon in the perfusion medium. The intracellular calcein fluorescence transiently changed because of the active fluorochrome efflux (Fig 4).

**Fig 5:**
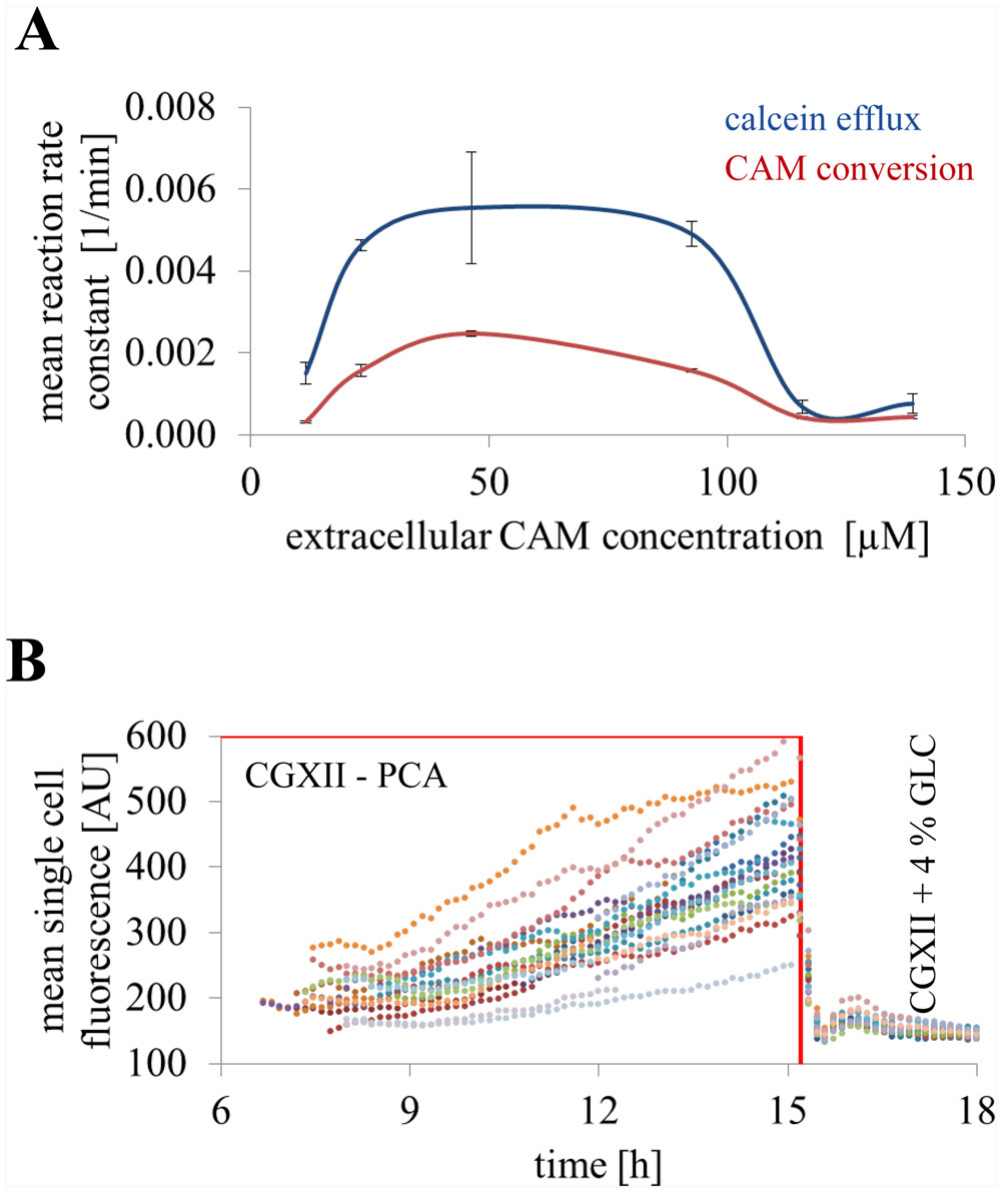
Mean CAM conversion rate constant and mean calcein efflux rate constant. **(A)** A mean maximal single cell reaction rate constant at 0.0025 min^-1^ was determined for CAM conversion under inhibition of calcein efflux due to carbon limitation. A maximal mean single cell calcein efflux rate constant was determined to be approximately twice as high at 0.005 min^-1^ (n = 5 colonies for each mean maximal single cell reaction rate constant). **(B)** Limitations in carbon supply inhibited cell division and energy driven transport of calcein out of the cell. Thus, mean single cell traces increase linearly over time until carbon supply was continued (every colored dotted line represent one individual cell, n = 15 cells).

Media shift experiments were performed to discriminate and determine the influence of calcein efflux. Mean single cell reaction rate constants of CAM conversion to calcein in dependency of the extracellular substrate concentration were determined immediately after the initiation of carbon depletion (Fig 5A). After 10 h of famine condition the medium supply was switched back to CGXII + 4% GLC. After the re-supply of glucose, cells responded with an immediate and significant reduction of intracellular mean single cell fluorescence under feast conditions (Figs 4 and 5B). The efflux rate constants of calcein secretion were determined in the initial 50 min of the reestablished feast condition (Fig 5A). The maximal mean single cell reaction rate constant at 0.005 min^-1^ of the calcein efflux was found to be twice as high as the maximal mean single cell reaction rate constant at approximately 0.0025 min^-1^ of the CAM conversion. However, *C*. *glutamicum* cells always showed remaining mean calcein fluorescence depending on their relevant enzyme activity, cell size, and environmental cultivation conditions.

Despite of the changes in mean single cell fluorescence, the resulting apparent growth rates at all five CAM concentrations showed no significant differences between all CAM concentrations. The apparent growth rates rapidly decreased after initiating carbon depletion and comparably recovered with an increased standard deviation after starvation stress (Fig 4).

### Experimental validation

**Fig 6:**
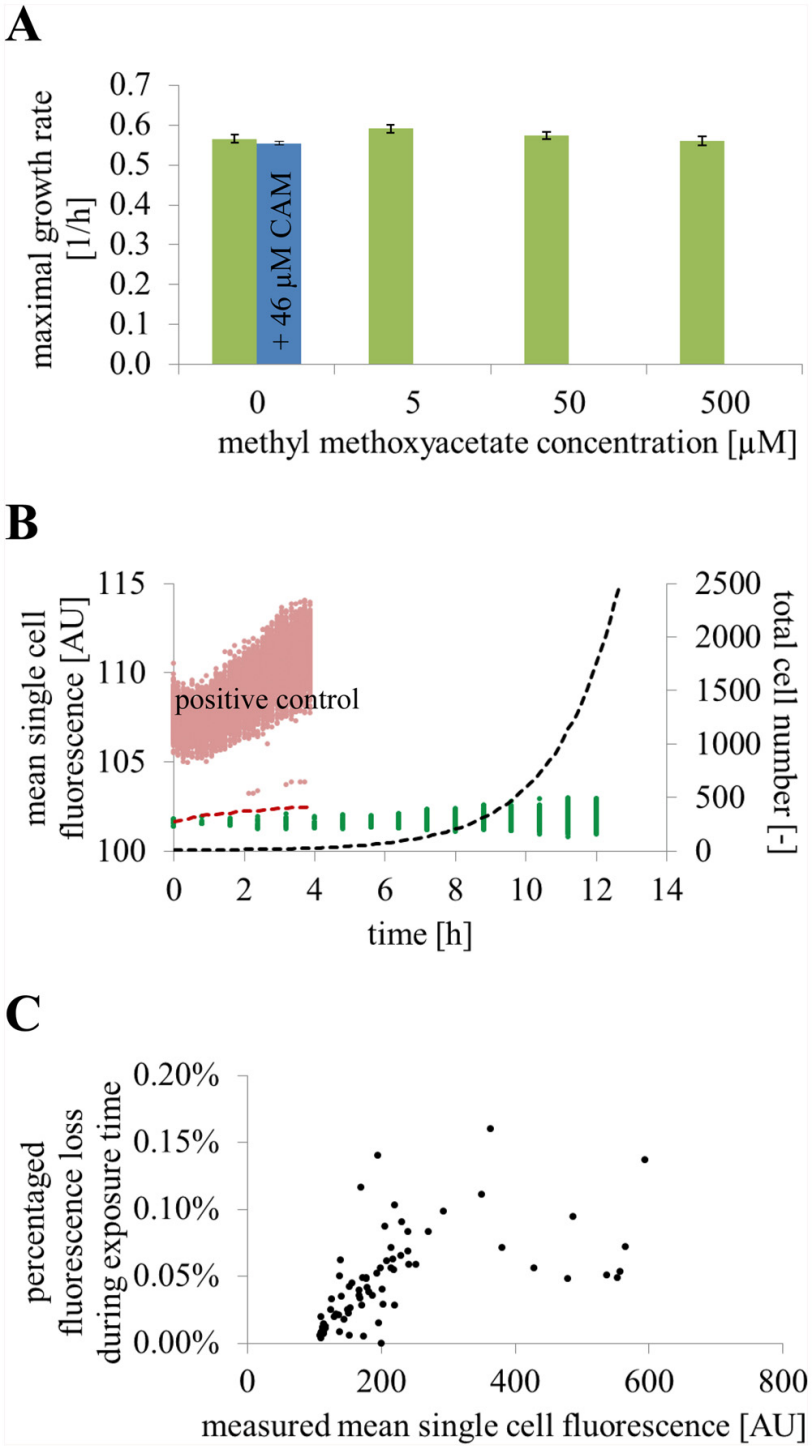
Experimental validation of the metabolic activity sensing method. **(A)** The CAM-surrogate methyl methoxyacetate was added to CGXII + 4% GLC and infused in three different concentrations in three separate supply channels of the microfluidic device to analyze the impact on growth by the intracellular digestion of the acetoxymethyl ester groups of CAM. The average maximal growth rates of five chambers cultivated with CGXII + 4% GLC with addition of three different methyl methoxyacetate concentrations are compared to a reference without addition (n = 5 colonies). The tested methyl methoxyacetate concentrations at 5 μM, 50 μM, and 500 μM corresponded to a CAM concentration of 1.7 μM, 16.7 μM, and 166.7 μM, respectively. In comparison the result for bacterial growth in CGXII + 4% GLC **+** 46 μM CAM is given. **(B)** An influence of the excitation light exposure (phototoxicity) during the fluorescence time lapse imaging was investigated by infusing DHCAM, which emits green fluorescence if light induced oxygen radical species are present. Typical experimental light exposure resulted in exponential cell growth (dashed black line, n = 5 colonies) and basal mean single cell fluorescence (green scatter plot, only every 6^th^ frame was measured, n = 5 colonies). In contrast, cells of the positive control experiment were initially exposed (> 1 sec) to maximal light intensity before starting time lapse imaging. Control cells (n = 271 cells) displayed immediate increase in the mean single cell fluorescence (pink scatter plot) and a stagnating total cell number (red dashed line) due to photo-oxidative stress. **(C)** Photobleaching was determined to be marginal as plotted as percentage of signal loss over mean single cell fluorescence (n = 85 cells).

The presented metabolic activity sensing was validated regarding non-invasiveness to bacterial growth and fluorescence signal stability. As already indicated by Fig 4, a mentionable impact on growth by CAM addition could not be found for concentrations up to 139 μM. Furthermore, a possible influence on growth by metabolized ester groups attached to CAM was analyzed by supplying the CAM-surrogate substrate methyl methoxyacetate. It is supposed to be taken up comparable to CAM and it is then enzymatically converted to ethanol and acetic acid. The methyl methoxyacetate concentrations were varied from 5 μM to 500 μM in CGXII + 4% GLC, corresponding to 1.7 μM to 166.7 μM CAM. No remarkable growth rate differences were observed between microcolonies grown in CGXII + 4% GLC with various methyl methoxyacetate concentrations or with addition of 46 μM CAM (corresponding to 138 μM methyl methoxyacetate) (Fig 6A).

Furthermore, phototoxicity due to fluorochrome exposure was analyzed by replacing CAM with the reduced dihydroxycalcein acetoxymethyl ester (DHCAM). DHCAM was taken up and was hydrolyzed comparable to CAM. In contrast, DHCAM requires oxidation by intracellular radical oxygen species (ROS) typically induced under photo-oxidative stress, to generate the green fluorescence of the probe calcein green. As described in literature, photo-oxidative stress by ROS, which were introduced by energetic light exposure, is responsible for intracellular damage of DNA, proteins, and cell membranes and triggers stress responses of microorganisms [36]. ROS were detected supplying DHCAM during the cultivation of *C*. *glutamicum* with CGXII + 4% GLC. For positive control, inoculated cells were additionally exposed (> 1 sec) to the highest illumination intensity before time lapse imaging to generate abundant intracellular ROS. Fig 6B shows that phototoxic stress was clearly indicated for the positive control by increased mean single cell fluorescence (pink scatter plot) and a stagnating total cell number (red dotted line). In contrast, the typical experimental illumination intensity during phase contrast and fluorescence imaging of calcein resulted in basal mean single cell fluorescence of calcein green (green scatter plot) and cell growth (black dotted line).

Concurrently, violet fluorescent calcein had a low vulnerability to photobleaching under standard experimental conditions with low light illumination. Photobleaching of calcein was determined with cells in a growth arrest phase due to carbon starvation. It was determined to reduce the mean single cell fluorescence by less than 0.2% at every fluorescence time lapse imaging snapshot (Fig 6C).

Besides, the excellent biological compatibility, calcein also performed no recognizable photobleeding. As evident from Fig 5B, calcein was contained intracellularly until the initiation of active transport by resupply of glucose. Moreover, calcein fluorescence showed a signal-to-noise ratio of 15:1 and higher compared to the extracellular medium. Nevertheless, significant increase of mean fluorescence of a colony resulted not of a mere growth reducing cultivation condition. However, it depends if bacterial growth is influenced due to reduction of the internal energy level due to increased ATPase activity (*e*.*g*. secretion of internal H^+^ excess) [35] or ATP depletion by substrate limitation [37].

### Metabolic activity sensing under intermittent nutrient limitation

**Fig 7:**
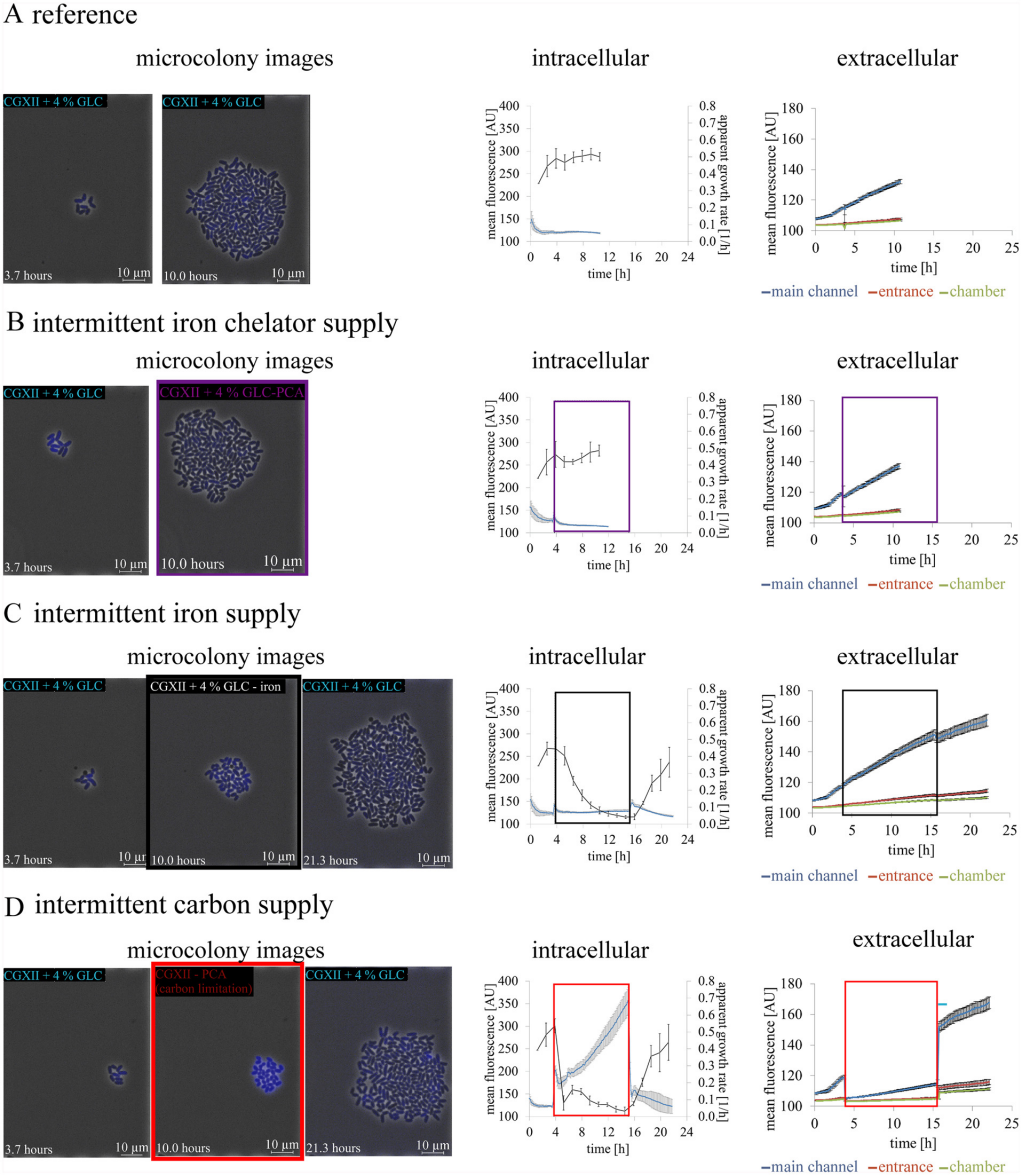
Metabolic activity sensing of *C*. *glutamicum* wild type at intermittent nutrient limitation in minimal medium CGXII. Bacterial cells were cultivated in minimal medium CGXII with 4% glucose (CGXII + 4% GLC) at pH 7 before an indicated shift to a depletion phase by switch of perfusion medium supply. **(A)** Reference cultivation under continuous supply of CGXII + 4% GLG. **(B-D)** After 4 h pre-cultivation, the microchambers were perfused with minimal medium (n = 10 colonies). **(B)** without iron chelator protocatechuate (PCA) (CGXII + 4% GLC—PCA, marked with violet boxes, n = 10 colonies), **(C)** without iron (CGXII + 4% GLC—iron, marked with black boxes, n = 10 colonies), and **(D)** with carbon limitation (CGXII—PCA, marked with red boxes, n = 10 colonies), respectively. For carbon and iron depletion conditions the medium was switched back to initial medium after 15 h. Microcolony images of every cultivation conditions are shown at different experimental time points. The mean fluorescence of the colony and apparent growth rate over time are shown in comparison to the extracellular mean fluorescence of the perfusion medium in the supply channel (10 μm fluid height), inside the cultivation chamber entrances (1 μm fluid height) and in the direct cell proximity (1 μm fluid height).

Hence, starvation stress was induced to experimentally manipulate the calcein fluorescence in *C*. *glutamicum*. Therefore, cells were pre-cultivated for 4 h under normal conditions (CGXII + 4% GLC). Then an intermittent supply of the iron chelator procatechuate (PCA) (Fig 7B), iron (Fig 7C) or glucose and procatechuate (carbon limitation) (Fig 7D) was established for 12 h. Full nutrient supply was only re-provided to iron depleted and carbon starved cells after 12 h. Subsequently, recovery and growth of bacterial cells was initiated again (Fig 7C and 7D). Cells exposed to iron and carbon depletion needed approximately 24 h to reach comparable cell numbers compared to 12 h during the reference cultivation under continuous supply of CGXII + 4% GLC (Fig 7A). A late depletion phase of PCA after 4 h showed only minor impact on mean fluorescence or growth (Fig 7B). In contrast the switch to limitation of iron (see S6 and S10 Videos) and carbon (see S11 and S12 Videos) was accompanied by cell growth stagnation, respectively. Furthermore, elongated cells were found after iron re-supply (see S10 Video) and under PCA limitation (S5 Video).

The apparent growth rate revealed that iron depletion provoked a considerable growth reduction and cell division was inhibited. *C*. *glutamicum* cells with a deficiency of carbon exhibited a remarkable decline of cell growth but continued cell division resulting in smaller descendants. Although the growth reduction was comparable during the starvation phase of iron limitation as well as carbon deprivation, the mean fluorescence differed significantly according to the missing medium component. The mean fluorescence homogeneously increased steadily for all cells in the absence of the major carbon source glucose and metabolizable PCA, whereas the mean fluorescence remained unchanged without iron supply (Fig 7C and 7D).

Further on, the extracellular calcein fluorescence of the perfusion medium was compared to the intracellular fluorescence. This extracellular fluorescence was measured at three positions in various replicates: i) inside the supply channels, ii) at the connection channels inside the chamber and iii) in close proximity to the cells. The fluorescence measurement inside the supply channel gave a sum signal of all chambers connected upstream resulting in a global signal of calcein efflux. Supply channel measurements revealed higher fluorescence than inside the cultivation chambers due to the tenfold medium height compared to the chamber height.

The extracellular mean fluorescence of the reference and the cultivation condition with intermittent iron chelator supply as well as iron deprivation, reflected our findings of the intracellular calcein continuously released into the surrounding medium (Fig 7A and 7B, respectively). However, under absence of glucose the course of the significantly lowered extracellular mean calcein fluorescence was contrasted to the continuously increasing intracellular mean fluorescence, proving calcein efflux inhibition (Fig 7D). Although the intracellular mean fluorescence remained rather constant during 12 h of iron starvation, calcein was continuously secreted and consequently CAM uptake and conversion proceeded steadily (Fig 7C).

**Fig 8:**
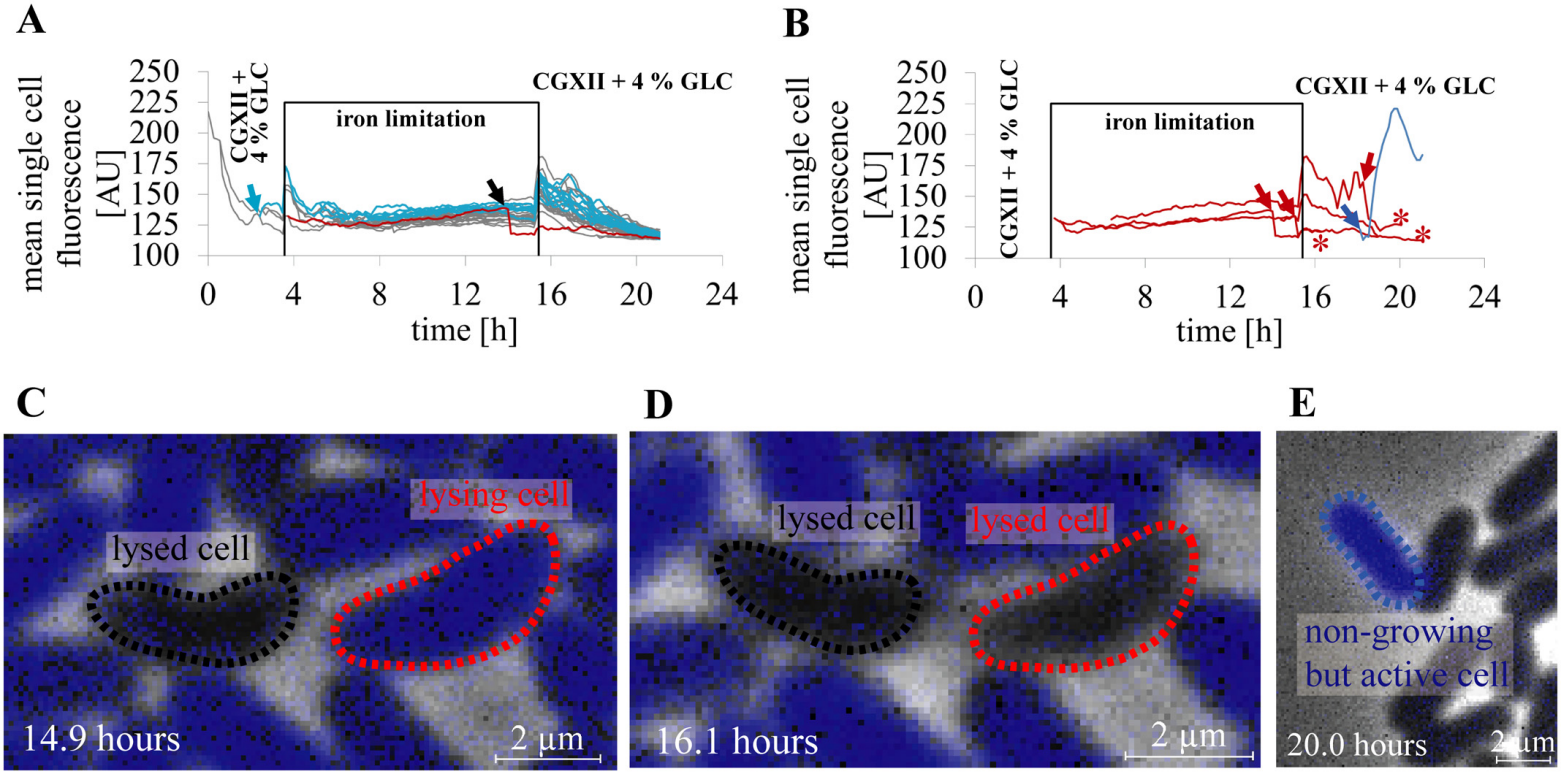
Metabolic activity sensing under iron limitation at single cell level. **(A)** Mean single cell fluorescence traces of all descendants of a progenitor cell are shown under intermittent iron supply. A daughter cell with higher calcein fluorescence than its siblings (light blue arrow) generated growing descendants with increased mean single cell fluorescence traces (light blue lines) in comparison to other descendants of the initial progenitor cell (grey mean single cell fluorescence traces). A single spontaneously non-growing cell changed from a dividing state to a non-growing state loosing esterase activity and intracellular calcein due to lysis (indicated by black arrow, lysed cell shown in **(C)** and **(D)**, respectively). **(B)** Mean single cell fluorescence traces of spontaneously non-growing cells of a microcolony are shown. Lysing cells (red lines) lost fluorescence spontaneously after lysis (red arrow). However, they were still detectable as apparently intact cells (end of recognition marked with red asterisks). The mean single cell fluorescence traces of a spontaneously non-growing but metabolically active cell (blue line) are shown in comparison. Mean single cell fluorescence increases shortly after cell birth (blue arrow). **(C)** A lysed but apparently intact cell (marked with black dashed line) and a cell directly before performing lysis (marked with red dashed line) are depicted. **(D)** Lysed cells still appear to be intact cells (red dashed line and black dashed line, respectively) after lysis. These non-growing cells showed no calcein fluorescence and were considered to be metabolically non-active. **(E)** A non-growing but metabolically active cell after re-supply of iron with elevated mean single cell fluorescence (marked by blue dashed line).

On a single cell basis, iron starvation caused heterogeneous phenotypes at the end of iron limitation and during the first five hours after iron resupply as illustrated in Fig 8 and S7 Video. The mean single cell traces of all growing and metabolically active descendants of one progenitor cells are shown in comparison with a spontaneously non-viable cell that lost its metabolic activity after lysis (Fig 8A). Bacteria showed an increase in their mean single cell fluorescence initially after media switch to CGXII + 4% GLC—iron and to CGXII + 4% GLC, respectively. The bacterial cells that were growth inhibited due to iron limitation showed comparable mean single cell fluorescence as their descendants after recovery during re-supply of iron.

As illustrated in Fig 3A, non-growing cells due to iron induced stress can be distinguished in i) metabolically active fluorescent cells and ii) non-viable cells with reduced mean single cell fluorescence, with experimental examples shown in Fig 8. In contrast to non-growing but active cells, we define non-viable cells as cells without the ability to restart growth and show no CAM conversion with resulting calcein fluorescence. The mean single cell fluorescence shortly increased after the medium switch before iron depletion and before the recovery phase, respectively. Cells with higher intracellular fluorescence than their siblings produced descendants with increased mean single cell fluorescence in comparison (Fig 8A, light blue lines). Although bacteria recovered and continued to grow after iron starvation (Fig 8A), a minority of cells exhibited a non-viable lysing phenotype (Fig 8B–8D, S8 Video) or despite of full nutrient availability, a non-growing but metabolically active state with remarkably increased mean single cell fluorescence (Fig 8B and 8E, and S9 Video).

**Fig 9:**
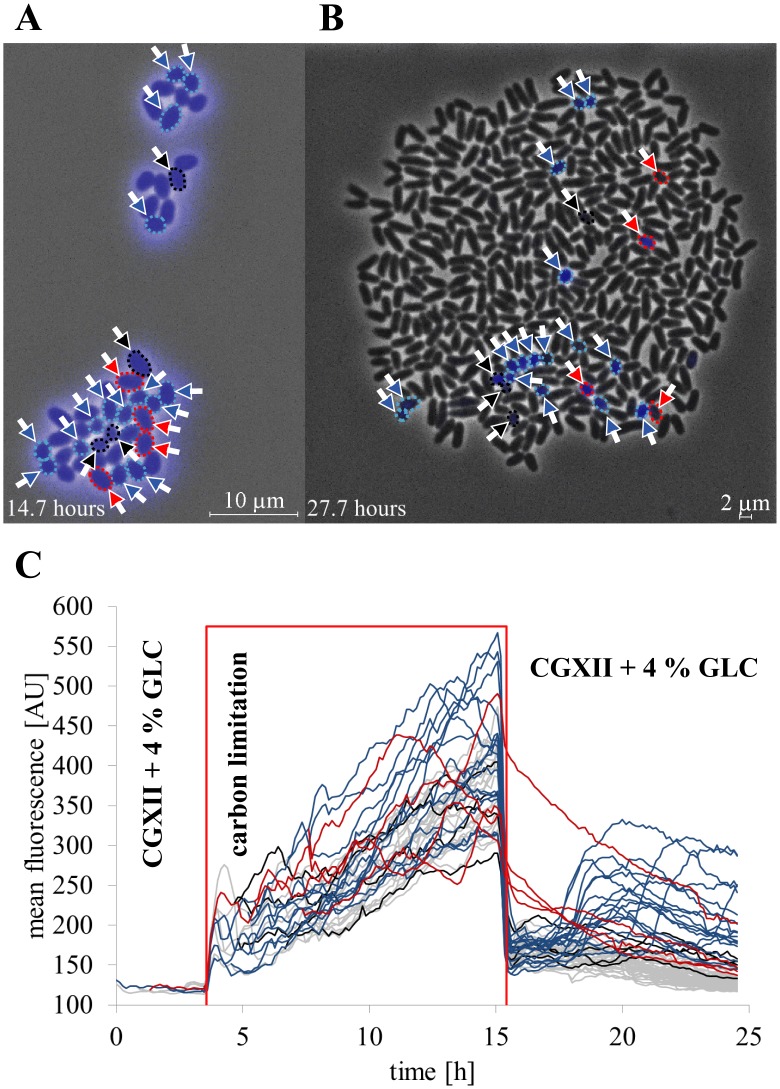
Metabolic activity sensing under carbon limitation at single cell level. **(A)** Microcolony image at the end of the cultivation phase under carbon limitation. Cells that performed no cell division after resupply of carbon are marked with arrows and are framed with dotted lines colored according to their mean single cell fluorescence traces in **(C). (B)** Microcolony image after regrowth under full nutrient supply at the end of cultivation. Cells that performed no cell division after carbon re-supply, are marked with arrows and are framed with dotted lines colored according to their mean single cell fluorescence traces in **(C). (C)** Mean single cell fluorescence traces of growing and non-growing bacteria before, during and after carbon depletion. Cells, which exhibit cell growth and division (grey lines), are compared to three phenotypes of non-growing cells with alternating mean single cell fluorescence during famine phase and reduced rate constants of calcein efflux under feast condition (red lines), increased mean single cell fluorescence during famine phase and after two hours after carbon resupply (blue lines) and average mean single cell fluorescence in comparison to normally dividing cells (black lines), respectively.

After returning to full carbon supply, few cells stayed in a non-growing and non-dividing, but metabolically active state. This rare phenotype was present in every analyzed cultivation chamber (n = 10 cultivation chambers). We observed that these dormant cells could be identified by their much higher mean single cell calcein fluorescence compared to their siblings during the metabolic activity sensing. The appearance and evolution of a considerably high number of those non-growing *C*. *glutamicum* cells in a colony is depicted in Fig 9 and shown in S12 Video. If carbon supply was changed from famine (Fig 9A, end of starvation phase) to feast (Fig 9B, end of cultivation) condition, most non-growing cells (blue and black lines) reacted with an immediate drastic decrease in the mean single cell fluorescence. This behavior was similar compared to readily growing cells with continued cell division. Some non-growing cells reacted with a delay of mean single cell fluorescence decrease after carbon re-supply.

Using the single cell mean fluorescence traces, the non-growing cells could be subdivided in different phenotypes as shown in Fig 9C. Almost every fifth cell of these dormant bacteria exhibited a slowed down decrease in the mean single cell fluorescence after re-supply of carbon (Fig 9, indicated red). The other two categories showed similar mean single cell fluorescence during the starvation phase, but differed after the recovery phase of the colony. On the one hand there were non-growing cells with unremarkable course of mean single cell fluorescence compared to growing cells (Fig 9, indicated black), on the other hand the majority of non-growing cells revealed an increase in the mean single cell fluorescence after some hours (Fig 9, indicated blue).

### Temporary growth inhibited *C*. *glutamicum* cells due to antibiotic exposure

It is actively discussed for severe diseases like tuberculosis and multidrug resistant pathogens, that there is an interfering relation between a transient state of reduced metabolic activity and antimicrobial tolerance or inheritable antibiotic resistance of cells [25,38–41]. The metabolic activity sensing based on CAM was used to compare the bacteriostatic antibiotic chloramphenicol (CHL) and the bacteriocidal ampicillin (AMP) in their impact on bacterial growth and metabolic activity. CHL inhibits the protein synthesis that impacts among other protein expression the bioneogenesis of enzymes such as esterases, whereas AMP targets the bacterial cell wall synthesis. Thus, the cell wall structure is altered impairing, *e*.*g*. cell growth or molecular containment.

**Fig 10:**
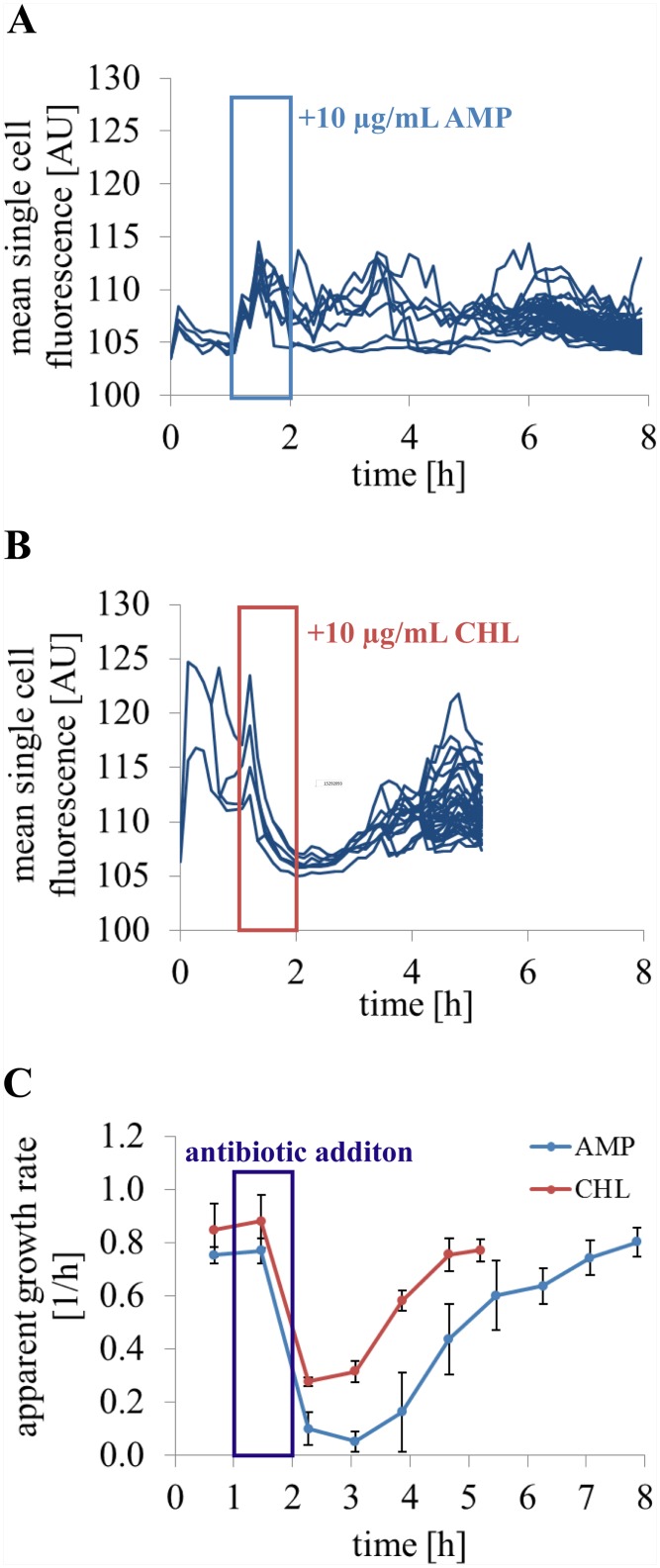
Metabolic activity sensing of cells exposed to unviable antibiotic concentrations and metabolic activity changes of descendants after antibiotic stress. After an initial growth phase in complex medium BHI, cells were exposed to **(A)** 10 μg/mL ampicillin (cell wall inhibition, n = 5 colonies) and **(B)** 10 μg/mL chloramphenicol (inhibition of protein synthesis, n = 5 colonies), respectively, for one hour. The antibiotics were added to the perfusion medium during the exposure time and after an hour perfusion with antibiotic free BHI was continued. Calcein mean single cell fluorescence revealed how the antibiotics change the bacterial fitness during antibiotic exposure. (**C**) The apparent growth rate was determined for five microcolonies treated for one hour with AMP or CHL.

Changes in calcein fluorescence of growing bacteria and their subsequently evolving descendants differed according to the applied antibiotic (Fig 10A and 10B, S3 and S4 Videos). Exponentially growing *C*. *glutamicum* cells were exposed to fatal concentration at 10 μg/mL of CHL and AMP, respectively, for one hour, as shown in Fig 10. Both antibiotic exposures were followed by an immediate arrest of cell growth (Fig 10C). The antibiotic CHL caused a faster change in mean single cell fluorescence compared to cells exposed to AMP addition. CHL impaired the growth and homogeneity of mean single cell calcein fluorescence of *C*. *glutamicum* cells and their descendants less than AMP at identical conditions (Fig 10A and 10B). After cells recovered from CHL stress, the mean single cell fluorescence increased simultaneously for all bacteria of the colony.

AMP caused an immediate increase in the mean single cell fluorescence. In addition, the growth totally stagnated during the antibiotic treatment of one hour and for the next subsequent two hours (Fig 10A and 10C). In comparison, cells recovered faster from the treatment with bacteriostatic CHL than from contact with bactericidal AMP (Fig 10C). Both antibiotics induced heterogeneous mean single cell fluorescence of subsequent populations after growth disturbance by these antibiotics. AMP additionally caused elongated as well as bifurcated cells. Further, asymmetric cell division happened frequently. Following the increased noise of mean single cell calcein fluorescence, the esterase activity and calcein efflux were not decreased significantly in average after AMP treatment, but increased in cell to cell variation. In contrast to CHL, some cells reduced their mean single cell fluorescence during AMP presence remarkably and entered a non-growing state.

## Discussion

### Non-invasive metabolic activity sensing

Intracellular calcein fluorescence is a universal indicator that the cell has active esterases and the energetic capability to perform transport mechanisms to secrete calcein. Therefore, comparative metabolic activity sensing relying on co-factor independent hydrolysis and fluorochrome trafficking could be demonstrated with intermittent deprivation of carbon, iron and the iron chelator PCA.

Fluorochrome incorporation, ester conversion, and light exposure were found to be non-invasive for the bacterial growth. The fluorescence signal was not found to be prone to photobleaching or photobleading. Both were not the reason for reduction of the mean single cell fluorescence following carbon re-supply after a starvation phase of 10–12 h. The revealed energy dependent calcein efflux of the *Actinobacteria* representative applied in the present work gives rise to the assumption that comparable ATPase activities are present to those reported for cancer cells [13,42]. The mechanism of CAM uptake or calcein secretion by prokaryotes is not completely understood yet, but could be of interest to use CAM turnover for toxicity assays or multidrug resistance screening as it is already done with cancer cells [13,42]. We performed a growth perturbation test with the antibiotics ampicillin and chloramphenicol, differing in mode of action, to demonstrate a temporal resolved insight on adaption of bacteria to abrupt antimicrobial exposure. After one hour of antibiotic addition the recovery and regrowth of these stressed bacteria and the impact on their descendants could be shown. The enhanced heterogeneity after ampicillin contact was supposed to be induced by disturbance of CAM uptake and calcein transport mechanism or calcein containment due to the antibiotic impact on cell wall synthesis. Chloramphenicol impaired mean single cell fluorescence especially during exposure and shortly after. This can be explained by rather reduced esterase activity than influence of calcein leakage.

Although, there are approaches with calcein green reported for physiology analysis of prokaryotes using FACS in literature [16,18], a comparable time resolved approach under required environmental stability as presented here and recommended for mammalian cells [13] has not been reported to our knowledge so far. Nevertheless, there are plenty methodologies of measuring the metabolic activity of prokaryotes. It has to be distinguished weather a quantitative knowledge of a bacterial population is required or comparative information of single cells is necessary. Invasive methods require quenching and/or cell lysis, *e*.*g*. intracellular enzyme activity measurement [43] or metabolic profiling [40]. These are analyses that provide snapshot insights in dependency of sampling time points. Moreover, these assays are generally very specific for enzymatic reactions and reveal precise information about metabolic pathways in detail. However, these analyses are often sophisticated and labor intensive. For measurements many cells have to be sampled, prepared and lysed. Thus, no information about dynamic changes of intracellular enzyme activity is provided as demonstrated with our comparative fluorescence method.

Another technical method to test presence of metabolic activity in environmental samples is isothermal microcalorimetry that is a precise measurement of generated heat by cellular reactions of metabolism [44] and 16s RNA (rRNA) detection by surface plasmon resonance imaging [45]. For both methods it can be stressed that these are real-time measurements, although the result interpretation can be challenging for non-experts [44,45].

Non-invasive metabolic activity sensing using bacterial substrates with stable isotopes is also of interest for diagnostic of human pathogens presence in patience. Approaches of stable-isotope breath tests using detection of isotopes of carbon, nitrogen or oxygen as evidence of infections are reviewed in [46]. In spite of the requirement, that the metabolic activity analysis has to be non-invasive to the host, the use of stable isotopes has to be performed by specialized scientist and has to be devoted with the relevant resources.

However, fluorescence based real-time measurement of metabolic activity enable scientists to determine physiological changes of bacteria in unviable cultivation conditions or metabolic activity changes due to substrate fluctuations. Metabolic key reactions used as detection mechanism are mostly based on redox reaction, respiratory activity, presence of rRNA, cellular energy pool or other enzymatic reactions as reviewed by Hammes et al. 2011 [47]. These metabolic activity measurements are either time resolved signals from bacterial suspensions or single cell resolved snapshots of endpoint reactions. Hence, the ideal prospective method of molecular resolved quantitative metabolic activity of viable and growing prokaryotes has still to be developed.

Even though, fluorophore expression system often based on GFP have a high potential to be advanced to single cell real-time monitoring of the metabolic activity, they share the disadvantages of expression heterogeneity and dependence of molecular oxygen for molecule ripening [38,48]. Furthermore, the species of interest has to be genetically modified beforehand.

Otherwise the use of dyes to prove enzymatic reactions require that the cellular uptake is given and neither product nor fluorogenic substrate cause toxicity for long time observations [49]. Further, differential cellular uptake of a fluorogenic substrate can contribute to heterogeneous fluorescence output and has to be taken into account. Nevertheless, finding an appropriate substrate for microbial metabolic activity sensing can be the needle in the haystack, but as examined with CAM and *C*. *glutamicum* can help to answer the key question active or not in many experimental conditions.

### Application of the metabolic activity sensing method

We applied the temporal resolved metabolic activity sensing based on intracellular fluorescence modulating enzyme reactions with microfluidic cultivation with lack of nutrient components and addition of antibiotics to demonstrate the applicability of the method with increasing impact on metabolic activity. Growth of *C*. *glutamicum* is reported by Liebl et al. 1989 to be stimulated by iron chelators as PCA in batch cultures with minimal medium. [37,50] Siderophore-mediated iron transport in *C*. *glutamicum* was postulated in literature, but the mechanism and function is not fully stated, yet [51]. Metabolic activity sensing and single cell growth determination showed no impact, if PCA is omitted after 4 hours pre-cultivation with full nutrient supply. PCA might be an initial growth stimulating factor.

The influence of iron depletion is more difficult to elucidate [52]. For our model organism *C*. *glutamicum* it is reported, that the regulator of iron homeostasis DtxR controls a range of genes directly or indirectly. These genes are related to, e.g., i) TCA cycle enzymes (aconitase, succinate dehydrogenase), ii) hydrogen peroxide decomposing catalase, iii) iron uptake, iv) and iron storage [52,53]. Iron is an important nutrient for growth and cell division, which were inhibited as long as iron was omitted in the perfusion medium. Nevertheless, CAM uptake, conversion and calcein efflux were not impaired by iron limitation. Metabolic activity sensing during intermittent iron starvation demonstrated that spontaneously non-growing cells can be distinguished due to intracellular calcein fluorescence in active cells and non-viables. Also cells appeared to be intact according phase contrast images, disintegrated cells can be identified by means of rapid mean single cell fluorescence loss due to cell lysis.

Non-growing cells that arose after intermittent carbon limitation proved to be metabolically active. These non-growing but metabolically active cells appeared in every analyzed microcolony under lack of carbon and differed phenotypically according to their mean single cell fluorescence after carbon re-supply. The resuscitation promoting factor Rpf2 is reported to trigger the regrowth of non-growing starved *C*. *glutamicum* cells after a switch from famine to feast condition. Rpf2 expression is controlled by GlxR, RamA, RamB in dependency of presence or absence of glucose. GlxR alone is involved in the control of 150 genes of carbon anabolism, catabolism and respiration of *C*. *glutamicum* [54,55]. The complex regulation of Rpf2 may explain the heterogeneity of dormant cells observed after carbon limitation. This convinced us to consider further research using metabolic activity sensing on non-growing and recovering cells. Clearly, studies of non-dividing bacteria are challenging due to overgrowth by readily growing viable cells [56]. Therefore, we consider the presented method as highly relevant for other bacteria reported developing dormant phenotypes as it is the case for *M*. *tuberculosis* that is closely related to *C*. *glutamicum* [24,25,38].

A tremendous increase in the mean fluorescence under carbon limitation supported the conclusion that calcein is secreted energy dependent, because in the absence of glucose intracellular ATP was reduced. Another hypothesis for the drastic change in mean fluorescence can be induction of esterase activity and enhanced calcein production. However, increasing extracellular CAM concentrations did not result in a constant increase of calcein fluorescence. Therefore, a constitutive intracellular esterase activity is applicable. Nevertheless, the calcein esterase activity of *C*. *glutamicum* has not been characterized yet and require more experimental insights *e*.*g*. by gene expression analysis.

The fluorogenic substrate CAM is conventionally used to validate viability of mammalian cells [13,14]. For cancer cells an energy-dependent transport of calcein by ATP-binding cassette (ABC) transporters like MRP-1 is reported in literature. These ATPases are related to multidrug resistance of tumour cells to several structurally unrelated cancer therapeutic drugs and were analysed by their potential to secrete calcein green [13,42,57]. The hydrolysis of CAM and the efflux of calcein of tumor cells is already demonstrated in literature and commercialized for mammalian viability testing or multidrug resistance potential of tumor cell lines [13]. *C*. *glutamicum* harbors ABC-type multidrug transport systems [58] and bears ABC-type multidrug transporter genes involved in homeostasis [35].

Nevertheless, revealing the molecular mechanism of calcein efflux has importance to further develop the metabolic activity sensing with CAM to a method of bacterial multidrug resistance screening as already established for tumor cells [13,42]. Thus, drug related bacterial ABC transport mechanism can be considered as a further important application of our metabolic activity sensing method in future. Since effects induced by antibiotics are already demonstrated in relation to mycobacterial ATP metabolism of non-growing and growing phenotypes to elucidate evolving antimicrobial resistance [59].

The use of metabolic activity sensing with CAM for antimicrobial testing was demonstrated with an exemplary use of antibiotics AMP and CHL. The antibiotics differ in mode of action and they generated different mean single cell fluorescence traces of descendants after antibiotic exposure. The increased heterogeneity of mean single cell calcein fluorescence under AMP addition is partly explained by the impaired cell wall integrity due to bacterial cell wall synthesis inhibition. Furthermore, resistance of *C*. *glutamicum* to AMP is influenced in contrast to CHL by the expression level of the potential multidrug resistance gene *cepA* encoding an efflux pump like protein [60].

Spatial and temporal resolution as given by the single cell microfluidic cultivation approach was advanced with non-invasive instantaneous fluorescence imaging. Thus, else hidden changes of metabolic activity after nutrient depletion or exposure to antimicrobials could be made visible. Also efflux of calcein is not fully understood, yet, the conversion from the one to the other is in combination with a sampling free microfluidic approach a powerful tool to gain new insights in the metabolic activity of growing and non-growing bacteria. Non-growing, dormant or resistant cells exhibit a large potential for bacterial survival of antimicrobial substances such as antibiotics.

## Supporting Information

S1 Video Metabolic activity sensing under reference conditions. The colony growth and calcein fluorescence is shown in comparison using the complex medium brain heart infusion (BHI, BD, Germany) and the minimal medium CGXII as described by Keilhauer et al. 1993 [29], respectively.
(MP4)

S2 Video Metabolic activity sensing at different media pH. Example colonies growing in BHI medium with the pH 6.6, pH 7.0 and pH 7.4 are given in comparison, respectively.
(MP4)

S3 Video Short term growth impairment by AMP. The impact on single cell growth and calcein fluorescence by short term exposure of antibiotics at a concentration of 10 μg/mL is shown for bacteriocidal ampicillin. The growth arrest and cell deforming effect of AMP was stronger compared to CHL at the same concentration. Non-viable cells were shrinking and fading.
(MP4)

S4 Video Short term growth impairment by CHL. The impact on single cell growth and calcein fluorescence by short term exposure of bacteriostatic chloramphenicol at a concentration of 10 μg/mL is shown for bacteriocidal ampicillin and.
(MP4)

S5 Video Intermittant iron chelator supply. After 3.7 hours, the perfusion medium was switched to procatechuate (PCA) free conditions (CGXII + 4% GLC—PCA).
(MP4)

S6 Video Intermittant iron supply. After 3.7 hours, the perfusion medium was switched to CGXII medium without iron (CGXII + 4% GLC—iron). The limitation of iron arrested the growth of *C*. *glutamiucum* cells until full media supply was returned after 15.2 hours.
(MP4)

S7 Video Intermittant iron supply with single cell events. After 3.7 hours, the perfusion medium was switched to without carbon (CGXII—PCA). The limitation of carbon arrested the growth of *C*. *glutamiucum* cells until full media supply was returned after 15.2 hours.
(MP4)

S8 Video Bursting iron depleted cells. Bursting cells only appeared after returning iron containing perfusion medium to iron depleted cells.
(MP4)

S9 Video Spontaneous non-growing cell after iron depletion. (MP4)

S10 Video Elongated cells after iron depletion. Under limitation conditions without iron chelator or after resupply of full nutrient supply following iron limitation a phenotype of elongated cells frequently could be observed.
(MP4)

S11 Video Intermittant carbon supply. (MP4)

S12 Video Non-growing cells after carbon depletion. (MP4)

## References

[1] Votyakova T V, Kaprelyants AS, Kell DB. Influence of Viable Cells on the Resuscitation of Dormant Cells in Micrococcus luteus Cultures Held in an Extended Stationary Phase: the Population Effect. 1994;60: 3284–3291. 10.1128/aem.60.9.3284-3291.1994 PMC201800 16349381

[2] Unge A, Tombolini R, Mølbak L, Jansson JK. Simultaneous Monitoring of Cell Number and Metabolic Activity of Specific Bacterial Populations with a Dual gfp-luxAB Marker System. 1999;65: 813–821. 10.1128/aem.65.2.813-821.1999 PMC91100 9925621

[3] Seletzky JM, Noack U, Fricke J, Hahn S, Büchs J. Metabolic activity of Corynebacterium glutamicum grown on L -lactic acid under stress. Appl Microbiol Biotechnol. 2006;72: 1297–1307. 16642330 10.1007/s00253-006-0436-0

[4] Tuson HH, Auer GK, Renner LD, Hasebe M, Tropini C, Salick M, et al Measuring the Stiffness of Bacterial Cells from Growth Rates in Hydrogels of Tunable Elasticity. Mol Microbiol. 2012;84: 18. 10.1111/j.1365-2958.2012.08063.x PMC3359400 22548341

[5] Besant JD, Sargent H, Kelley SO. Rapid electrochemical phenotypic profiling of antibiotic-resistant bacteria. Lab on a Chip. Royal Society of Chemistry; 2015;15: 2799–2807. 10.1039/c5lc00375j 26008802

[6] Gómez-Sjöberg R, Leyrat AA, Pirone DM, Chen CS, Quake SR. Versatile, Fully Automated, Microfluidic Cell Culture System. Anal Chem. 2007;79: 8557–63. 17953452 10.1021/ac071311w

[7] Liu D, Evans T, Zhang F. Applications and advances of metabolite biosensors for metabolic engineering. Metabolic engineering. Elsevier; 2015;31: 35–43. 10.1016/j.ymben.2015.06.008 26142692

[8] Stephens PJ, Hewitt CJ, Powell JR, Badley RA, Nebe-von-Caron G. Analysis of Bacterial Function by Multi-Colour Fluorescence Flow Cytometry and Single Cell Sorting. J Microbiol Meth. 2000;42: 97–114. 10.1016/s0167-7012(00)00181-0 11000436

[9] Burke HM, Gunnlaugsson T, Scanlan EM. Recent advances in the development of synthetic chemical probes for glycosidase enzymes. Chemical Communications. Royal Society of Chemistry; 2015;51: 10576–10588. 10.1039/c5cc02793d 26051717

[10] Orenga S, James AL, Manafi M, Perry JD, Pincus DH. Enzymatic substrates in microbiology. Journal of Microbiological Methods. 2009;79: 139–55. 10.1016/j.mimet.2009.08.001 19679151

[11] Rotman B, Papermaster BW. Membrane Properties of Living Mammalian Cells As Studied by Enzymatic Hydrolysis of Fluorogenic Esters. Biochemistry-US. 1965;55: 134–141. 10.1073/pnas.55.1.134 PMC285766 5220862

[12] Haugland RP, MacCoubrey IC, Moore PL. Dual-Fluorescence Cell Viability Assay Using Ethidium Homodimer and Calcein Am. United States patent US005314805A; 5,314,805, 1994. p. 12.

[13] Byrd TF, Hoang LT, Kim EG, Pfister ME, Werner EM, Arndt SE, et al The microfluidic multitrap nanophysiometer for hematologic cancer cell characterization reveals temporal sensitivity of the calcein-AM efflux assay. Science Report. 2014;4: 10. 10.1038/srep05117 PMC4038811 24873950

[14] Poulsen CR, Culbertson CT, Jacobson SC, Ramsey JM. Static and dynamic acute cytotoxicity assays on microfluidic devices. Analytical chemistry. 2005;77: 667–72. 15649069 10.1021/ac049279i

[15] Lichtenfels R, Biddison WE, Schulz H, Vogt AB, Martin R. OF CARE-LASS (calcein-release-assay), an improved fluorescence-based test system to measure cytotoxic T lymphocyte activity. J Immunol Methods. 1994;172: 227–39. 7518485 10.1016/0022-1759(94)90110-4

[16] Diaper JP, Edwards C. The use of fluorogenic esters to detect viable bacteria by flow cytometry. Journal of Applied Bacteriology. 1994;77: 221–228.

[17] Joux F, Lebaron P. Use of fluorescent probes to assess physiological functions of bacteria at single-cell level. Microbes and infection. 2000;2: 1523–35. 11099939 10.1016/s1286-4579(00)01307-1

[18] Comas J, Vives-Rego J. Enumeration, viability and heterogeneity in Staphylococcus aureus cultures by flow cytometry. Journal of Microbiological Methods. 1998;32: 45–53.

[19] Hammes F, Egli T. Cytometric methods for measuring bacteria in water: advantages, pitfalls and applications. Analytical and bioanalytical chemistry. 2010;397: 1083–95. 10.1007/s00216-010-3646-3 20352197

[20] Shapiro HM, Ave H, Newton W. Microbial analysis at the single-cell level : tasks and techniques. J Microbiol Meth. 2000;42: 3–16. 10.1016/s0167-7012(00)00167-6 11000426

[21] Halldorsson S, Lucumi E, Gómez-Sjöberg R, Fleming RMT. Advantages and challenges of microfluidic cell culture in polydimethylsiloxane devices. Biosens Bioelectron. Elsevier; 2015;63: 218–31. 10.1016/j.bios.2014.07.029 25105943

[22] Young JW, Locke JCW, Elowitz MB. Rate of Environmental Change Determines Stress Response Specifity. PNAS. 2013;110: 4140–4145. 10.1073/pnas.1213060110 23407164 PMC3593889

[23] Hartmann M, Barsch ÆA, Niehaus ÆK, Andreas Æ, Jo TÆ. The glycosylated cell surface protein Rpf2, containing a resuscitation-promoting factor motif, is involved in intercellular communication of Corynebacterium glutamicum. Arch Microbiol. 2004;182: 299–312. 15480574 10.1007/s00203-004-0713-1

[24] Evangelopoulos D, da Fonseca JD, Waddell SJ. Understanding anti-tuberculosis drug efficacy: rethinking bacterial populations and how we model them. Int J Infect Dis. International Society for Infectious Diseases; 2015;32: 76–80. 10.1016/j.ijid.2014.11.028 25809760

[25] Gengenbacher M, Kaufmann SHE. Mycobacterium tuberculosis: success through dormancy. FEMS Microbiology Reviews. 2012;36: 514–32. 10.1111/j.1574-6976.2012.00331.x 22320122 PMC3319523

[26] Grünberger A, Probst C, Helfrich S, Nanda A, Stute B, Wiechert W, et al Spatiotemporal microbial single-cell analysis using a high-throughput microfluidics cultivation platform. Journal of Cytometry A. 2015; 10.1002/cyto.a.22779 26348020

[27] Westerwalbesloh C, Grünberger A, Stute B, Weber S, Wiechert W, Kohlheyer D, et al Modeling and CFD simulation of nutrient distribution in picoliter bioreactors for bacterial growth studies on single-cell level. Lab on a Chip. Royal Society of Chemistry; 2015; 10.1039/C5LC00646E 26345659

[28] Grünberger A, Probst C, Heyer A, Wiechert W, Frunzke J, Kohlheyer D. Microfluidic picoliter bioreactor for microbial single-cell analysis: fabrication, system setup, and operation. Journal of visualized experiments: JoVE. 2013;82: 50560 10.3791/50560 24336165 PMC4044959

[29] Keilhauer C, Eggeling L, Sahm H. Isoleucine Synthesis in Corynebacterium glutamicum: Molecular Analysis of the ilvB-ilvN-ilvC Operon. J Bacteriol. 1993;175: 9. 10.1128/jb.175.17.5595-5603.1993 PMC206616 8366043

[30] Schneider CA, Rasband WS, Eliceiri KW. NIH Image to ImageJ: 25 years of image analysis. Nature Methods. Nature Publishing Group; 2012;9: 671–675. 10.1038/nmeth.2089 PMC5554542 22930834

[31] Schindelin J, Arganda-Carreras I, Frise E, Kaynig V, Longair M, Pietzsch T, et al Fiji: an open-source platform for biological-image analysis. Nature methods. 2012;9: 676–82. 10.1038/nmeth.2019 22743772 PMC3855844

[32] Helfrich S, Azzouzi CE, Probst C, Seiffarth J, Grünberger A, Wiechert W, et al Vizardous: Interactive Analysis of Microbial Populations with Single Cell Resolution. 2015; 10–12. 10.1093/bioinformatics/btv468 26261223

[33] Marienfeld S, Uhlemann E, Schmid R, Krämer R, Burkovski A. Ultrastructure of the Corynebacterium glutamicum cell wall. Antonie van Leeuwenhoek. 1997;72: 291–297. 9442270 10.1023/a:1000578811089

[34] Carlo D Di, Aghdam N, Lee LP. Single-Cell Enzyme Concentrations, Kinetics, and Inhibition Analysis Using High-Density Hydrodynamic Cell Isolation Arrays. Analytical Chemistry. 2006;78: 4925–4930. 16841912 10.1021/ac060541s

[35] Follmann M, Ochrombel I, Krämer R, Trötschel C, Poetsch A, Rückert C, et al Functional genomics of pH homeostasis in Corynebacterium glutamicum revealed novel links between pH response, oxidative stress, iron homeostasis and methionine synthesis. BMC genomics. 2009;10: 10.1186/1471-2164-10-621 PMC2807442 20025733

[36] Ziegelhoffer EC, Donohue TJ. Bacterial responses to photo-oxidative stress. Nat Rev Microbiol. 2009;7: 856–863. 10.1038/nrmicro2237 19881522 PMC2793278

[37] Unthan S, Grünberger A, van Ooyen J, Gätgens J, Heinrich J, Paczia N, et al Beyond growth rate 0.6: What drives Corynebacterium glutamicum to higher growth rates in defined medium. Biotechnology and bioengineering. 2014;111: 359–71. 10.1002/bit.25103 23996851

[38] Manina G, Dhar N, McKinney J. Stress and host immunity amplify Mycobacterium tuberculosis phenotypic heterogeneity and induce nongrowing metabolically active forms. Cell Host Microbe. 2015;17: 32–46. 10.1016/j.chom.2014.11.016 25543231

[39] Bhargava P, Collins J. Boosting Bacterial Metabolism to Combat Antibiotic Resistance. Cell Metab. 2015;21: 154–155. 10.1016/j.cmet.2015.01.012 25651168

[40] Peng B, Su Y, Li H, Han Y, Guo C, Tian Y, et al Exogenous Alanine and/or Glucose plus Kanamycin Kills Antibiotic-Resistant Bacteria. Cell Metab. 2015;21: 249–261. 10.1016/j.cmet.2015.01.008 25651179

[41] Dhar N, Dubée V, Ballell L, Cuinet G, Hugonnet J-E, Signorino-Gelo F, et al Rapid cytolysis of Mycobacterium tuberculosis by faropenem, an orally bioavailable β-lactam antibiotic. Antimicrobial agents and chemotherapy. 2015;59: 1308–19. 10.1128/AAC.03461-14 25421469 PMC4335862

[42] Feller N, Broxterman HJ, Währer DCR, Pinedo HM. ATP-dependent efflux of calcein by the multidrug resistance protein (MRP): no inhibition by intracellular glutathione depletion. FEBS Lett. 1995;368: 385–388. 7628644 10.1016/0014-5793(95)00677-2

[43] Wang C, Cai H, Zhou Z, Wan H. Metabolic Analysis of a Corynebacterium glutamicum IdhA Mutant During an Efficient Succinate Production Using pH-Control Under Oxygen Deprivation In: Zhang T-C, Nakajima M, editors. Advances in Applied Biotechnology. Berlin, Heidelberg: Springer; 2015 pp. 375–387.

[44] Braissant O, Bachmann a., Bonkat G. Microcalorimetric assays for measuring cell growth and metabolic activity: Methodology and applications. Methods. Elsevier Inc.; 2015;76: 27–34. 10.1016/j.ymeth.2014.10.009 25461776

[45] Foudeh AM, Trigui H, Mendis N, Faucher SP, Veres T, Tabrizian M. Rapid and specific SPRi detection of L. pneumophila in complex environmental water samples. Analytical and bioanalytical chemistry. 2015;407: 5541–5. 10.1007/s00216-015-8726-y 25935681

[46] Timmins GS. Detecting virulence and drug-resistance mycobacterial phenotypes in vivo. Trends in microbiology. Elsevier Ltd; 2015;23: 321–3. 10.1016/j.tim.2015.02.013 PMC4458167 25800730

[47] Hammes F, Berney M, Egli T. Cultivation-independent Assessment of Bacterial Viability. Adv Biochem Engin Biotechnol. 2011;124: 123–150. 10.1007/10_2010_95 21069588

[48] Waidmann MS, Bleichrodt FS, Laslo T, Riedel CU. Bacterial luciferase reporters: the Swiss army knife of molecular biology. Bioengineered bugs. 2011;2: 8–16. 10.4161/bbug.2.1.13566 21636983

[49] Ullrich S, Karrasch B, Hoppe H, Jeskulke K, Mehrens M. Toxic effects on bacterial metabolism of the redox dye 5-cyano-2,3-ditolyl tetrazolium chloride. Applied and environmental microbiology. 1996;62: 4587–93. 16535471 10.1128/aem.62.12.4587-4593.1996 PMC1389009

[50] Liebl W, Klamer R, Schleifer K-H. Requirement of Chelating Compounds for the Growth of Corynebacterium glutamicum in Synthetic Media. Appl Microbiol Biotechnol. 1989;32: 205–210.

[51] Dertz EA, Stintzi A, Raymond KN. Siderophore-Mediated Iron Transport in Bacillus subtilis and Corynebacterium glutamicum. J Biol Inorg Chem. 2006;11: 1087–1097. 16912897 10.1007/s00775-006-0151-4

[52] Wennerhold J, Krug A, Bott M. The AraC-type Regulator RipA Represses Aconitase and Other Iron Proteins from Corynebacterium under Iron Limitation and Is Itself Repressed by DtxR. Journal of Biological Chemistry. 2005;280: 40500–40508. 16179344 10.1074/jbc.M508693200

[53] Brune I, Werner H, Hüser AT, Kalinowski J, Pühler A, Tauch A. The DtxR protein acting as dual transcriptional regulator directs a global regulatory network involved in iron metabolism of Corynebacterium glutamicum. BMC Genomics. 2006;7: 21 16469103 10.1186/1471-2164-7-21 PMC1382209

[54] Schröder J, Tauch A. Transcriptional regulation of gene expression inCorynebacterium glutamicum: the role of global,masterand local regulators in the modularand hierarchical gene regulatory network. FEMS Microbiol Rev. 2010;34: 685–737. 10.1111/j.1574-6976.2010.00228.x 20491930

[55] Jungwirth B, Emer D, Brune I, Hansmeier N, Alfred P, Eikmanns BJ, et al Triple transcriptional control of the resuscitation promoting factor 2 (rpf2) gene of Corynebacterium glutamicum by the regulators of acetate metabolism RamA and RamB and the cAMP-dependent regulator GlxR. FEMS Microbiol Lett. 2008;281: 190–197. 10.1111/j.1574-6968.2008.01098.x 18355281

[56] Keep NH, Ward JM, Cohen-Gonsaud M, Henderson B. Wake up! Peptidoglycan lysis and bacterial non-growth states. Trends Microbiol. 2006;14: 271–276. 16675219 10.1016/j.tim.2006.04.003

[57] Essodaı M, Broxterman HJ. Kinetic Analysis of Calcein and Calcein—Acetoxymethylester Efflux Mediated by the Multidrug Resistance Protein and P-Glycoprotein. 1998;2960: 2243–2250. 10.1021/bi9718043 9485370

[58] Schluesener D, Fischer F, Kruip J, Rögner M, Poetsch A. Mapping the Membrane Proteome of Corynebacterium glutamicum. Proteomics. 2005;5: 1317–1330. 15717325 10.1002/pmic.200400993

[59] Maglica Z, Özdemir E, McKinney JD. Single-Cell Tracking Reveals Antibiotic-Induced Changes in Mycobacterial Energy Metabolism. mBio. 2015;6: 1–11. 10.1128/mBio.02236-14 PMC4338811 25691591

[60] Sim S, Hong E, Kim Y, Lee H. Analysis of cepA Encoding an Efflux Pump-like Protein in Corynebacterium glutamicum. 2014;52: 278–283. 10.1007/s12275-014-3461-1 24535744

